# Risk for opioid misuse in chronic pain patients is associated with endogenous opioid system dysregulation

**DOI:** 10.1038/s41398-021-01775-z

**Published:** 2022-01-12

**Authors:** Javier Ballester, Anne K. Baker, Ilkka K. Martikainen, Vincent Koppelmans, Jon-Kar Zubieta, Tiffany M. Love

**Affiliations:** 1 Department of Psychiatry, University of Utah, Salt Lake City, UT USA; 2 Mental Health Addiction Services, VA Salt Lake City Health Care System, Salt Lake City, UT USA; 3 Department of Anesthesiology, Duke University, Durham, NC USA; 4 Department of Radiology, Medical Imaging Center, Tampere University Hospital, Tampere, Finland; 5 Department of Psychiatry, Northwell Health, John T. Mather Memorial Hospital, Port Jefferson, NY USA

**Keywords:** Addiction, Molecular neuroscience, Predictive markers

## Abstract

µ-Opioid receptors (MOR) are a major target of endogenous and exogenous opioids, including opioid pain medications. The µ-opioid neurotransmitter system is heavily implicated in the pathophysiology of chronic pain and opioid use disorder and, as such, central measures of µ-opioid system functioning are increasingly being considered as putative biomarkers for risk to misuse opioids. To explore the relationship between MOR system function and risk for opioid misuse, 28 subjects with chronic nonspecific back pain completed a clinically validated measure of opioid misuse risk, the Pain Medication Questionnaire (PMQ), and were subsequently separated into high (PMQ > 21) and low (PMQ ≤ 21) opioid misuse risk groups. Chronic pain patients along with 15 control participants underwent two separate [^11^C]-carfentanil positron emission tomography scans to explore MOR functional measures: one at baseline and one during a sustained pain-stress challenge, with the difference between the two providing an indirect measure of stress-induced endogenous opioid release. We found that chronic pain participants at high risk for opioid misuse displayed higher baseline MOR availability within the right amygdala relative to those at low risk. By contrast, patients at low risk for opioid misuse showed less pain-induced activation of MOR-mediated, endogenous opioid neurotransmission in the nucleus accumbens. This study links human in vivo MOR system functional measures to the development of addictive disorders and provides novel evidence that MORs and µ-opioid system responsivity may underlie risk to misuse opioids among chronic pain patients.

## Introduction

Chronic pain is a serious, prevalent, worldwide health problem [1]. Chronic nonspecific back pain is the most common chronic pain syndrome, impacting around 38% of the global population [2]. Opioids remain the frontline treatment for chronic pain conditions, contributing to what the Centers for Disease Control and Prevention have declared to be an opioid epidemic. In concert with a steady rise in prescription opioids, nonmedical use of these medications has also increased, with opioids now reportedly the most commonly abused drugs in the country [3]. In 2019, 9.7 million people (3.5%) aged 12 or older had misused prescription pain relievers in the past year [4]. Orthopedic pain (34.8%) was the primary reason for an opioid prescription, followed by dental conditions (17.3%), back pain (14.0%), and headache (12.9%) [5]. Nearly one-third of chronic pain patients endorse opioid misuse behaviors [6], but not every chronic pain patient prescribed opioids develops a problematic pattern of use. Accordingly, identifying individuals who are at risk for opioid misuse prior to beginning opioid therapy is of significant clinical value. Unfortunately, the processes which underlie enhanced misuse and addiction risk are not currently understood.

While multiple neurotransmitter systems likely contribute to the risk of misuse of pain medications, opioid systems are of particular interest. Activation of the endogenous mu-opioid receptor (MOR) system has been long known to reduce both sensory and affective responses to pain and stress [e.g. [7, 8]], and disruptions in this system are suspected to be involved in the pathogenesis of chronic pain. Human and animal studies indicate substantial interindividual variations in MOR activation and/or endogenous opioid system function. Such differences are further influenced by chronic pain [9–16], introducing varying capacity to respond to exogenous opioid treatment or perhaps even a predisposition to develop persistent pain.

The MOR neurotransmitter system also mediates the reinforcing and hedonic effects of both natural and artificial rewards [17–21]. MORs robustly modulate activity within the mesolimbic pathway—a critical reward circuit consisting of dopamine neuron projections from the ventral tegmental area (VTA) to the nucleus accumbens (NAc) [22]. Activation of MORs located on VTA GABAergic interneurons produces hyperpolarization [23, 24]. This results in disinhibition of DA neurons projecting to the NAc, causing enhanced NAc dopamine release and concomitant increases in reward learning and seeking [see review, [25]]. MOR activation contributes to drug reinforcement and addiction processes, where enhanced MOR activity is associated with heightened drive, increased consumption, and enhanced hedonic reactivity to reward [25].

The present study examines the role of MOR-mediated neurotransmission in risk for prescription opioid misuse in humans. Previous reports have investigated the effects of opioid medications on the MOR system in persons with opioid use disorders (OUD) [26, 27], which likely include adaptations of MOR signaling as a result of chronic opioid use and/or binge/deprivation patterns of use; however, no study to date has examined an at-risk population without current OUD. Here, we utilized [^11^C]-carfentanil positron emission tomography (PET) with the radiotracer [11C]carfentanil, a selective μ-opioid receptor radioligand [28] to examine relationships between baseline MOR concentrations (non-displaceable binding potential, BP_ND_) and endogenous opioid release in response to a standardized sustained pain challenge, as a function of misuse risk. Misuse risk was calculated from the Pain Medication Questionnaire (PMQ)—a clinical screening instrument used to assess potential for opioid medication misuse in the context of chronic pain [29]—in a sample of chronic nonspecific back pain patients. We initially hypothesized that individuals at high risk for opioid misuse would exhibit higher levels of baseline MOR BP_ND_, possibly secondary to chronic of lower endogenous opioid function in regions (i.e., NAc, amygdala) previously shown to be dysregulated in chronic pain [15] and further associated with addiction [30].

## Methods and materials

### Participants

We studied 28 chronic pain patients (11 males, 17 females, mean ± SD age, 38.0 ± 10.1) recruited from a pain clinic, and 15 healthy controls (HC; 6 males, 9 females, age 40.5 ± 9.5) recruited via advertisement. [^11^C]-carfentanil PET data from 16 chronic pain patients have been reported previously [15] and reanalyzed here. All participants were right-handed, non-smoking adults. Patients were excluded if they endorsed lifetime substance dependence, current nicotine dependence, or use of antipsychotics, stimulants, or recreational drugs. Power analyses examining group differences in MOR BP_ND_ between persons with fibromyalgia and controls indicated *n* = 27 would have 90% power to detect an effect at α = 0.05. All participants provided written informed consent. All procedures adopted were approved by the Investigational Review Board and Radioactive Drug Research Committee at the University of Michigan.

### Intake measures

#### Pain-related measures

Chronic pain subjects’ pain experiences were assessed using the McGill Pain Questionnaire (MPQ, [31]), a self-report measure used to quantify the sensory and affective qualities of pain and the Brief Pain Inventory (BPI, [32]), which characterizes pain severity and impact of pain on daily life. As pain fluctuates day to day, patients were asked to complete the BPI each day for a week. These data were averaged and used to compute pain severity and interference. For patients taking prescription opioids (*n* = 14), morphine milligram equivalent (MME) for total daily opioid dose was calculated using CDC guidelines (Table 1).

**Table 1:** Chronic back pain characteristics.

Measure	PMQ-H	PMQ-L	*t*	*p*
Pain intensity	56.9 ± 21.7	58.0 ± 24.0	0.12	0.90
Pain unpleasantness	62.0 ± 21.9	60.3 ± 26.0	0.18	0.86
MPQ sensory	10.9 ± 6.2	14.2 ± 7.3	1.27	0.22
MPQ affective	2.0 ± 2.3	1.9 ± 2.4	0.15	0.88
BPI severity^a^	5.5 ± 1.8	5.3 ± 1.8	0.31	0.76
BPI interference^a^	4.6 ± 1.9	4.8 ± 2.4	0.16	0.87
MME	22.7 ± 43.0	18.5 ± 22.2	0.33	0.74

Mean ± 1 SD of psychophysical measures at baseline.

^a^1 subject from the PMQ-H & PMQ-L groups failed to provide BPI data.

#### Opioid misuse risk

Opioid misuse risk was assessed prior to scanning using the PMQ, a 26-item self-report measure with good internal consistency and test-retest reliability in the context of persistent pain [33]. The PMQ has demonstrated superior predictive utility out of 14 similar assessment tools [34]. Individuals with high, relative to low, PMQ scores exhibit greater risk of OUD and likelihood to prematurely request opioid prescription refills [35]. Patients with PMQ scores >21 were classified as high risk (PMQ-H, *n* = 13), and those with PMQ scores ≤21 were grouped as low risk (PMQ-L, *n* = 15) [35].

#### Personality

Participants completed the Zuckerman-Kuhlman Personality Questionnaire (ZKPQ) prior to scanning to assess dimensions of impulsivity (ZKPQ-Impulsivity), a tendency to act without planning, and sensation seeking (ZKPQ-Sensation Seeking), a preference for novelty and excitement, which have both been previously associated with risk taking [36]. Three PMQ-L participants did not complete the ZKPQ and were excluded from these analyses.

### Neuroimaging

*PET*. [^11^C]-carfentanil data were acquired with a Siemens HR + scanner (Knoxville, TN). Radiotracer synthesis, image acquisition, and preprocessing protocols have been described in detail elsewhere [7, 37, 38]. Briefly, fifty percent of the [^11^C] carfentanil dose was administered as a bolus, and the remaining 50% as a continuous infusion using a computer-controlled pump to achieve steady-state tracer levels. The total activity of [^11^C]-carfentanil administered during each scan was 15.6 ± 0.6 mCi with a mass injection of <0.03 μg/kg per scan (Table 2). PET data were corrected for decay, attenuation, and motion [39]. A modified Logan graphical analysis [40] using the occipital cortex as a reference region was used to transform dynamic image data on a voxel-by-voxel basis into two sets of parametric maps: a tracer transport measure (K1 ratio) and a measure of receptor availability in vivo (non-displaceable binding potential, BP_ND_). The Logan method together with the bolus-continuous infusion allows for linearity in the plot typically 5–7 min after radiotracer administration, allowing for the calculation of BP_ND_ values early during scanning. Data obtained from 45 to 90 min post-tracer administration was utilized for the analyses presented here, as previously described [7, 15, 41, 42].

**Table 2:** Psychophysiological measures.

Scan	Measure	Controls	PMQ-H	PMQ-L	*F*	*p*
Baseline scan						
	[^11^C]-carfentanil injected dose (mCi)	15.9 ± 0.5	15.4 ± 0.6	15.7 ± 0.4	2.98	0.06
	[^11^C]-carfentanil mass injected (μg/kg)	0.01 ± 0.01	0.01 ± 0.01	0.01 ± 0.005	0.48	0.62
	PANAS positive	32.5 ± 6.6	29.5 ± 6.9	27.7 ± 9.2	1.45	0.25
	PANAS negative	11.5 ± 2.0	15.0 ± 3.6	14.1 ± 5.5	3.05	0.06
	POMS-TMD	−3.0 ± 10.2	23.8 ± 19.7	15.9 ± 18.9	9.76	<0.001
Pain scan						
	[^11^C]-carfentanil injected dose (mCi)	15.4 ± 0.5	15.4 ± 1.2	15.6 ± 0.4	0.36	0.70
	[^11^C]-carfentanil mass injected (μg/kg)	0.02 ± 0.01	0.01 ± 0.01	0.01 ± 0.01	0.89	0.42
	ΔPANAS positive	8.8 ± 7.7	3.7 ± 3.0	5.3 ± 10.3	1.36	0.27
	ΔPANAS negative	0.4 ± 1.9	0.9 ± 3.0	−1.2 ± 8.5	0.52	0.60
	ΔPOMS-TMD	−8.4 ± 11.3	8.8 ± 16.4	−0.4 ± 21.5	3.02	0.06
	Pain intensity	30.2 ± 17.0	42.73 ± 13.9	43.9 ± 23.0	2.16	0.13
	Pain unpleasantness	35.8 ± 30.5	46.4 ± 26.1	45.0 ± 24.4	0.57	0.57
	MPQ sensory	17.8 ± 8.0	16.2 ± 7.4	18.9 ± 7.7	0.39	0.68
	MPQ affective	1.5 ± 2.1	1.8 ± 2.8	1.9 ± 2.2	0.11	0.89
	Average 15-sec VAS	26.1 ± 13.5	39.5 ± 10.3	32.6 ± 16.3	2.79	0.08

Mean ± 1 SD of psychophysical measure of pain during the pain-stress challenge. VAS intensity refers to the average ratings of momentary pain acquired every 15 s for the duration of the pain-stress challenge (20 min). The remainder of the scales (MPQ; PANAS; and POMS-TMD) were obtained immediately after completion of the pain-stress challenge.

For spatial normalization, T1-weighted magnetic resonance imaging (MRI) scans were obtained on a General Electric (GE) Sigma 3 T scanner or GE Discovery 3 T scanner (number of slices = 154; voxel resolution = 1 mm^3^; flip angle = 15°; FOV = 250 × 260mm^2^; TR = 9.2 ms; TE = 1.9 ms). MRI and PET images were coregistered to each other using SPM12 (www.fil.ion.ucl.ac.uk/spm/) and normalized into Montreal Neurological Institute (MNI) space using the advanced normalization tools (ANTs) software package [43]. PET data were then smoothed with a 6-mm FWHM Gaussian kernel.

#### Pain-stress challenge

Participants underwent two randomized and counterbalanced PET scans: a pain scan, where participants experienced moderate levels of sustained pain over 20 min, and a baseline scan. This challenge has been described in detail previously [7, 15, 42]. Briefly, pain was induced via infusion of hypertonic saline (5%) into the left masseter muscle 45-min post-radiotracer administration. Every 15 s, participants rated their pain on a VAS scale of 0 (no pain) to 100 (most intense pain imaginable) and the infusion rate was adjusted via a computer-controlled closed-loop system targeting an average VAS rating of about 40 [44, 45]. At the completion of the pain challenge, subjects completed the MPQ to describe their experimentally-induced pain. Prior to scanning and immediately after the pain challenge, participants completed the Positive and Negative Affectivity Schedule (PANAS, [46]), and the Profile of Mood States (POMS, [47]) to changes in affective state and expressed as percent change. One subject in the PMQ-L group did not complete their pain scan and two subjects in both the PMQ-H and control group experienced technical problems and their data could not be utilized, resulting in *n* = 38 for pain analyses.

#### Image analysis

**Fig. 1:**
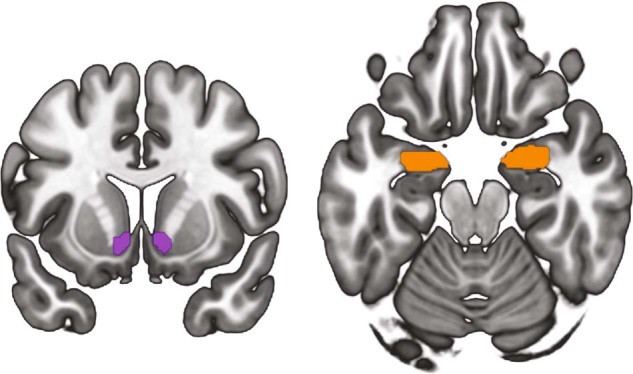
Regions of interest. Location of regions of interest. Visualizations were created with MRIcroGL (http://www.cabiatl.com/mricrogl/).

We used an a priori region of interest (ROI) approach to explore differences in MOR-related measures between groups. We focused on the NAc and amygdala due to their corresponding functionality in pain, negative affect and, more broadly, their prominent roles in addiction [7, 25, 30, 48, 49]. Additionally, alterations in MOR activity within these regions have been previously described in both animal and human models of addiction [e.g. [50, 51]]. ROIs were defined using the Harvard-Oxford atlas (http://www.cma.mgh.harvard.edu/) using a probability threshold of 25% (see Fig. 1).

Two measures of endogenous opioid system function were examined: Baseline MOR BP_ND_ and pain-induced changes in MOR BP_ND_, defined as the percent change in MOR BP_ND_ from baseline to the pain stress condition. The latter measure, reflects processes associated with endogenous opioid release, such as competition between the radiotracer and endogenous neurotransmitter and reduction in receptor affinity after the activation of MORs by the endogenous ligand [7, 8, 15].

We also conducted whole-brain exploratory analyses to determine whether we could detect group differences in baseline BP_ND_ or in pain-induced changes in MOR BP_ND_ outside our a priori defined regions of interest. We examined the effects of group on baseline and pain-induced changes to MOR BP_ND_ by applying a general linear model on a voxel-by-voxel basis using SPM12 (Wellcome Department of Cognitive Neurology, London, United Kingdom) for Matlab (MathWorks, Natick, Massachusetts). A cluster-forming threshold of *p* < 0.001 and a peak level significance threshold of *p* < 0.05 FWE (family-wise error) with a minimum cluster size of 10 voxels was used to define significant peaks.

### Statistical analysis

All statistical analyses were conducted using SPSS 26 (Armonk, NY, USA). Correlations between MOR BP_ND_ and changes in psychophysical measures were calculated using Kendall’s Tau-b coefficients due to the skewed distribution of these measures [52]. Comparisons between groups were performed using one-way analysis of variance (ANOVA) and *t*-tests. ROI analyses were corrected for multiple comparisons using a Bonferroni correction, with a significance threshold to *p* = 0.0125 to account for the 4 ROIs.

## Results

There were no significant differences in age (mean ± SD: Control, 40.5 ± 9.5, PMQ-H, 33.9 ± 10.3, PMQ-L 41.5 ± 8.6) between groups (*F*_(2,40)_ = 2.7, *p* = 0.08). Baseline clinical pain characteristics including pain severity, pain intensity and pain interference, and morphine equivalents are shown in Table 1. The number of individuals being prescribed opioids (*χ*^2^_(1,28)_ = 1.3, *p* = 0.26) also did not significantly differ between PMQ-H and PMQ-L groups.

A one-way ANOVA revealed significant group differences in ZKPQ-Sensation Seeking (*F*_(2,37)_ = 4.19, *p* = 0.02) with Tukey’s post-hoc comparisons indicating significantly higher ZKPQ-Sensation Seeking in PMQ-H compared to PMQ-L (*p* = 0.03), but only a trending toward significance when compared to the HC group (*p* = 0.08). However, there were no significant group differences in ZKPQ-Impulsivity observed (*F*_(2,37)_ = 1.07, *p* = 0.35).

As it may be expected that opioid misuse risk scores may track ZKPQ-Sensation Seeking and ZKPQ-Impulsivity scores, correlations between ZKPQ-Sensation Seeking, ZKPQ-Impulsivity and PMQ scores were examined. There were no significant relationships detected between PMQ scores and ZKPQ-Impulsivity (*τ*_b_ = 0.16, *p* = 0.30); however, a significant positive relationship was observed for ZKPQ-Sensation Seeking (*τ*_b_ = 0.40, *p* = 0.01).

### Baseline MOR BP_ND_ in chronic pain patients and controls

#### PMQ

**Fig. 2:**
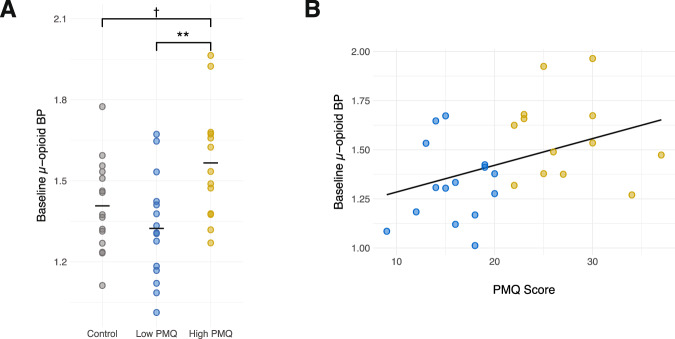
Regional differences in Mu-opioid receptor binding between individuals at high and low opioid misuse risk. **A** Significant differences in baseline MOR BP_ND_ between groups was obtained in the right amygdala, with individuals in the high-risk for opioid misuse group (PMQ-H) exhibited the highest mu-opioid receptor MOR BP_ND_. **B** Significant relationships between opioid misuse risk scores and baseline µ-opioid BP were also observed.

A one-way ANOVA showed significant differences in MOR BP_ND_ in the right amygdala (*F*_(2,40)_ = 5.58, *p* = 0.01), with Tukey’s post-hoc comparisons indicating the PMQ-H group exhibited higher BP_ND_ compared to the PMQ-L group (*p* = 0.01) and HC group (*p* = 0.09) (see Fig. 2). A similar trend was noted in the left amygdala (*F*_(2,40)_ = 2.98, *p* = 0.06). Consistently, we also noted a significant positive correlation between PMQ scores and MOR BP_ND_ in the same region (right amygdala, *τ*_b_ = 0.31, *p* = 0.02; left amygdala, *τ*_b_ = 0.34, *p* = 0.01), indicating higher baseline MOR availability was associated with higher risk for opioid misuse. No group differences in NAc MOR BP_ND_ were noted (right NAc: *F*_(2,40)_ = 1.41, *p* = 0.26; left NAc: *F*_(2,40)_ = 0.28, *p* = 0.76).

**Fig. 3:**
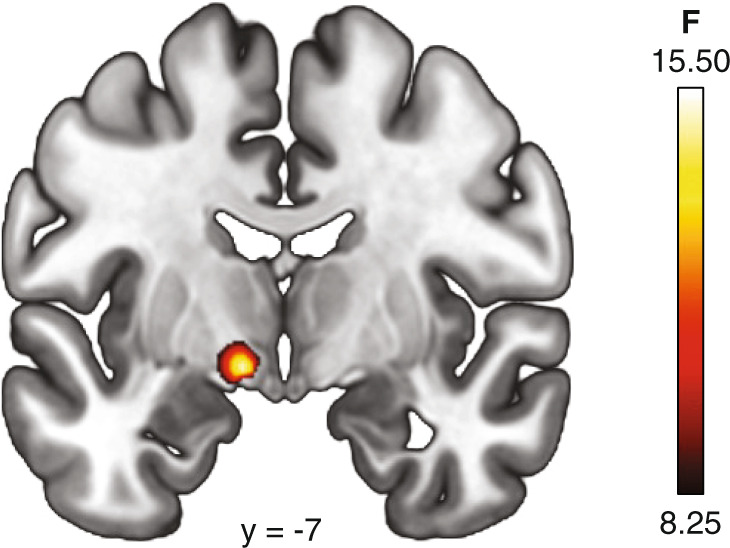
Whole-brain group differences in Mu-opioid receptor binding potential. Significant differences (*p*_FWE_ < 0.05) in baseline MOR BP_ND_ between groups was observed in the left extended amygdala, with individuals in the low-risk for opioid misuse group (PMQ-L) exhibiting lower mu-opioid receptor availability relative to PMQ-H and Control groups.

Exploratory whole-brain analyses revealed significant regional effects of group within an area corresponding to the left extended amygdala (*x*, *y*, *z* coordinates, −10, −6, −8; *F* = 15.72, cluster size = 90 mm^3^; *p*_FWE_ = 0.04, Fig. 3) in line with the results of our ROI analyses. No other regional differences as baseline were noted. Post-hoc analyses in SPSS revealed significantly lower MOR BP_ND_ in the PMQ-L group relative to both the PMQ-H group (*p* < 0.001) and the Control group (*p* = 0.003). No differences were observed between PMQ-H and Control groups (*p* = 0.163).

#### Affect

Both PMQ-H (*p* < 0.001) and PMQ-L (*p* = 0.01) groups showed significantly higher POMS-TMD scores than HC participants (*F*_(2,40)_ = 9.76, *p* < 0.001). These differences are unsurprising given the historic association between mood disturbance and chronic pain. No significant group differences in pre-scan positive affect were observed (*F*_(2,40)_ = 1.45, *p* = 0.25) though there was a trend (*F*_(2,40)_ = 3.05, *p* = 0.06) for higher negative affect among PMQ-H participants relative to controls (Table 2). There were no significant relationships detected between any of the affective measures and binding.

#### Back pain

Significant negative relationships between MPQ sensory back pain and MOR BP_ND_ in the right amygdala (*τ*_b_ = −0.29, *p* = 0.03) and left amygdala (*τ*_b_ = −0.31, *p* = 0.02) were observed. No other significant relationships were observed between MOR BP_ND_ and back pain intensity, unpleasantness, or among MPQ affective ratings.

#### Personality

There were no significant relationships between ZKPQ-Sensation Seeking and baseline MOR availability for any group. We did, however, observe significant positive relationships between ZKPQ-Impulsivity and MOR availability within the left NAc (*τ*_b_ = 0.29, *p* = 0.02), right NAc (*τ*_b_ = 0.31, *p* = 0.01), left amygdala (*τ*_b_ = 0.27, *p* = 0.03), and a trend in the right amygdala (*τ*_b_ = 0.22, *p* = 0.06).

### Pain stress-induced changes in MOR BP_ND_

#### PMQ

A one-way ANOVA indicated group differences in the capacity to activate endogenous opioid neurotransmission in response to the pain stressor (*F*_(2,35)_ = 4.30, *p* = 0.02), with Tukey’s post-hoc comparisons indicating MOR release was lower in the PMQ-L group than in the HC group in the left NAc (*p* = 0.03), however, this was not significant after our stringent Bonferroni correction for multiple comparisons (*p* < 0.0125). There was a trend towards a positive relationship between PMQ scores and pain-induced changes in MOR BP_ND_ within the left NAc (*τ*_b_ = 0.28, *p* = 0.05). No other group differences or relationships between PMQ scores and pain-induced changes in MOR BP_ND_ were noted.

We did not observe any significant effects of group within our whole-brain pain analyses. However, though not significant after correction for multiple corrections, we did observe a peak within an area corresponding to the extended amygdala (*x*, *y*, *z* coordinates, −10, −4, −8; *F* = 11.39, cluster size = 27mm^3^; *p*_uncorrected_ < 0.001).

#### Affect

There were no significant group differences in the pain-induced changes in POMS-TMD (*F*_(2,35)_ = 0.94, *p* = 0.40), positive affect (*F*_(2,35)_ = 0.15, *p* = 0.86), or negative affect (*F*_(2,35)_ = 0.83, *p* = 0.44) scores. We detected negative relationships between ΔPOMS-TMD and pain-induced changes in MOR BP_ND_ in the left amygdala (*τ*_b_ = −0.27, *p* = 0.02), left NAc (*τ*_b_ = −0.24, *p* = 0.04). A negative relationship between ΔPANAS negative affect and MOR release within the left NAc was also observed (*τ*_b_ = 0.29, *p* = 0.01). Hence, increases in distress and negative affect during the pain stress challenge were associated with lower endogenous opioid release. No other relationships between change in affect and MOR release were observed.

#### Back pain

No significant relationships were observed between pain-induced changes in MOR BP_ND_ and back pain intensity, unpleasantness, or MPQ ratings.

#### Experimental pain

We then examined the relationships between pain-induced changes in MOR BP_ND_ and experimental pain ratings. One-way ANOVAs indicated no significant group differences in pain intensity (*F*_(2,35)_ = 2.16, *p* = 0.13), pain unpleasantness (*F*_(2,35)_ = 0.57, *p* = 0.57), average 15-sec VAS ratings (*F*_(2,35)_ = 2.79, *p* = 0.08), or MPQ sensory (*F*_(2,35)_ = 0.39, *p* = 0.68) or MPQ affective (*F*_(2,35)_ = 0.11, *p* = 0.89) ratings following the pain challenge. There were no significant relationships between any of the experimental pain measures and pain-induced changes in MOR BP_ND_.

#### Personality

Consistent with the baseline findings, there were no significant relationships between ZKPQ-Sensation Seeking or ZKPQ-Impulsivity and pain-stress-induced change in MOR availability for any group.

## Discussion

The present study provides the first evidence in humans that risk for opioid misuse is associated with variations in endogenous opioid functional measures. Here we observe that individuals at high risk to misuse opioids (PMQ-H) have higher MOR BP_ND_ at rest within regions implicated in emotion and reward processing relative to individuals at lower risk (PMQ-L). Further, that following a standardized pain stressor, lower levels of endogenous opioid release were observed among individuals in the PMQ-L group, relative to controls, within the NAc, however, this difference did not reach our stringent a priori significance threshold. We also show that subjective pain experiences and emotional states are distinctly related to in vivo measures of MOR neurotransmission. Among chronic pain patients, back pain severity ratings were inversely related to baseline MOR BP_ND_, while enhanced negative affect during the pain stressor was related to blunted endogenous opioid system activity. These data may yield insight into the means by which chronic pain and dysregulated pain processing are reciprocally related to one another, and for the first time elucidate the neurobiological validity of a subjective measure of risk for opioid misuse.

The amygdala has long been recognized to play a significant role in pain regulation. MORs in the amygdala have been shown to inhibit nociceptive signaling in preclinical models [53, 54], and activation of endogenous opioid, MOR-mediated neurotransmission in the amygdala is associated with reductions in pain ratings in healthy human subjects [7, 38]. Previous research has posited that regional deficits in MOR-mediated neurotransmission are likely contributors to insufficient endogenous opioid pain control, amplified acute pain sensitivity, and risk for development of additional chronic pain disorders [15, 55, 56]. Indeed, we find both lower MOR availability among the PMQ-L patients relative to controls, as well as significant inverse relationships between self-reported sensory back pain symptoms and amygdala MOR BP_ND_ among chronic pain patients. Interestingly, in our ROI analyses, we noted significant differences between groups within the right amygdala but only a trend within the left amygdala. While the origin of such hemispheric differences is not known, there are well described functional asymmetries in the processing of negative emotions within the amygdala, with the right side playing a more prominent role [57, 58]. In addition, hemispheric asymmetries in MOR BP_ND_ have been previously noted in large scale PET studies with the amygdala and a NAc displaying higher MOR BP_ND_ within the left hemisphere [59].

While lower MOR BP_ND_ has been associated with deficient pain control, higher MOR BP_ND_ has been associated with risk-taking phenotypes such as high impulsivity and low deliberation [42]. In part, higher MOR BP_ND_ among PMQ-H patients may be associated with risk for substance misuse as a function of the opioid system’s influence over preferences for immediate rewards. In humans, greater preference for immediate monetary rewards and greater discounting of probabilistic gains and losses [60] is noted among individuals with opioid addictions relative to non-addicted individuals [61–63]—a preference which is exacerbated following mild opioid deprivation [64]. Preclinical models in rodents have demonstrated dose-dependent increases in delay discounting rates [65] and impulsive responding to the five-choice serial reaction time task [66] following morphine administration, while studies in mice have revealed reductions in motor impulsivity [67] and in perseverative responses to obtain reward [68] among MOR knockouts. Here, we observed the highest MOR BP_ND_ among those at highest risk to misuse opioids and among those reporting the highest levels of impulsivity but, interestingly, we did not observe any relationships between PMQ scores and impulsivity. This implies these constructs may be capturing distinct information about addiction and misuse vulnerability. But these data, along with previous work [42], demonstrate that even when using unique measures of risk, enhanced risk for substance misuse and addiction is related to endogenous opioid system functioning.

A comparatively lower magnitude pain-induced change in MOR BP_ND_ was observed in the left NAc among patients in the low risk group. The endogenous opioid system is heavily implicated in both pain and stress [e.g. [7, 38]]. Several lines of evidence indicate lower MOR reactivity in response to a painful challenge reflects lower capacity to effectively regulate responses to stress and may partially underlie the pathophysiology of chronic pain [15]. Here, we find that endogenous opioid activation of MOR-mediated neurotransmission in response to the pain challenge was relatively, though not significantly, blunted in the PMQ-L group relative to controls; an effect not observed in the PMQ-H group. These effects were noted in the absence of significant group differences in either sensory or affective responses to the challenge, indicating that while pain-induced endogenous opioid system activity varied, the experience of pain was similar across groups. We also noted a trend towards a positive relationship between PMQ scores and change in MOR BP_ND_ within the left NAc. This suggests that the capacity to activate the endogenous opioid system in response to pain may be an overlapping but also distinctive biomarker of chronic pain and risk for opioid misuse. Finally, we noted inverse relationships between total mood disturbance, negative affect, and endogenous opioid system activation by the experimental challenge, within the left NAc, indicating greater attenuation of negative affect is associated with greater recruitment of the MOR system during the challenge. This is consistent with previous PET studies that have shown MOR system activation in this region is involved in the modulation of not only pain, but also human affective responses [7, 38].

The present study provides the first evidence in humans that risk for opioid misuse is related to interindividual variations in the function of the endogenous opioid system. While previous PET studies have reported alterations in MOR system markers among persons with addictive disorders, including individuals with nicotine dependence [69], alcohol dependence [70], and gambling disorders [71], the present findings are the first to report altered endogenous opioid activity in persons at *risk* for opioid misuse. A few limitations should be noted in consideration of these results. First, these data were derived from a sample of non-neuropathic pain patients and the results may not be generalizable to populations with other forms of clinical pain, such as generalized muscular (e.g., fibromyalgia) or neuropathic pain. Second, the sample size is relatively small and cross-sectional, however well-controlled through specialized pain clinics. Future studies with larger, more heterogeneous samples will allow for more nuanced examinations of causal effects and aid in unpacking the unique contributions of psychological and neurobiological variables to risk for opioid misuse in the context of persistent pain. Additionally, our chronic pain sample included individuals at risk for opioid misuse, but did not include persons with a history of opioid misuse. Prevalence of opioid misuse within chronic pain populations is estimated to be as high as 29% [72]. It will be important for future investigations to determine how MOR signaling varies according not only to misuse risk but also according to misuse status. Despite these limitations, the present study contributes novel findings to the existing body of literature on neurobiological features of risk for opioid misuse.
